# Seasonal changes in objectively measured sedentary behavior and physical activity in Japanese primary school children

**DOI:** 10.1186/s12889-016-3633-5

**Published:** 2016-09-13

**Authors:** Chiaki Tanaka, John J. Reilly, Maki Tanaka, Shigeho Tanaka

**Affiliations:** 1 Division of Integrated Sciences, J. F. Oberlin University, 3758 Tokiwamachi, Machida, Tokyo, 194-0294 Japan; 2 Physical Activity for Health Group, School of Psychological Sciences and Health, University of Strathclyde, 50 George Street, Glasgow, G1 1QE Scotland UK; 3 Department of Child Education, Kyoto Seibo College, 1 Taya-cho, Fukakusa, Fushimi-ku, Kyoto 612-0878 Japan; 4 Department of Nutritional Science, National Institute of Health and Nutrition, National Institutes of Biomedical Innovation, Health and Nutrition, 1-23-1 Toyama, Shinjuku-ku, Tokyo, 162-8636 Japan

**Keywords:** Activity pattern, Summer vacation, Accelerometry

## Abstract

**Background:**

The recent prevalence of obesity in Japanese children is much higher compared to 1980. The present study compared daily sedentary behavior (SB) and physical activity (PA) between the school year and summer vacation in Japanese primary school children.

**Methods:**

Participants were 98 Japanese boys (8.9 ± 1.8 years at baseline) and 111 girls (9.1 ± 1.8 years). SB and PA were measured in May (school term) and July/August (summer vacation), 2011. SB and PA were assessed using a triaxial accelerometer (Active style Pro HJA-350IT, Omron Healthcare) for 7 consecutive days. The average number of minutes spent in SB (no more than 1.5 metabolic equivalents (METs)), light intensity activity (LPA; more than 1.5 to less than 3.0 METs) and moderate-to-vigorous physical activity (MVPA; 3.0 METs or more), and step counts were calculated for each individual. Moreover, the determinants/moderators of changes in SB and PA were examined.

**Results:**

Daily SB was significantly higher in the summer vacation than in the school year for both boys and girls (*p* < 0.05). Ambulatory and total LPA and MVPA, non-ambulatory LPA and step counts were lower in summer vacation in both genders (*p* < 0.001). Moreover, non-ambulatory MVPA was significantly lower in the summer vacation than in the school year for girls (*p* < 0.001). The decrease in non-ambulatory MVPA in boys and increase in SB in girls were significantly lower in those who participated in sports compared to those who did not (*p* < 0.040 or *p* < 0.033). The change in SB for boys was significantly associated with having a TV in the bedroom (*p* < 0.022).

**Conclusions:**

These findings show that primary school children in Japan are less active in the summer vacation, as indicated by both higher SB and lower LPA and ambulatory MVPA in both genders. Moreover, the seasonal change in non-ambulatory MVPA for Japanese children was affected by gender. This study also suggests that sports participation and bedroom TV ownership may moderate seasonal changes in PA and SB. The results emphasize the need to take summer vacation into account when planning interventions aimed at decreasing SB or increasing PA in Japanese children.

**Electronic supplementary material:**

The online version of this article (doi:10.1186/s12889-016-3633-5) contains supplementary material, which is available to authorized users.

## Background

The recent prevalence of obesity in Japanese children is much higher compared to 1980 [1]. Previous studies reported that overweight and obese Japanese children experienced greater weight gain during the summer vacations than during the school months [2, 3]. A maximum temperature of over 35 degrees in summer is typical in Japan every year [4]. Periods of high temperatures in summer may reduce the likelihood of children being physically active (PA) or may increase sedentary behavior (SB). Recently, Lewis et al. (2016) reported that daily maximum temperature was significantly associated with moderate-to-vigorous PA (MVPA) and SB time in primary school-aged children in Australia and Canada [5]. Moreover, children’s activity levels tend to be lower on weekdays compared to weekend days in the school year [6–8]. Identification of specific seasons that are characterized by low PA levels and/or high periods of SB is important for the design future public health interventions aimed at promoting PA and reducing SB, but summer vacation changes in PA and SB in Japanese children are poorly understood at present.

SB is distinct from PA [9–11], and it is possible for an individual to spend an excessive proportion of time in SB, even if they meet PA guidelines [12]. A recent review suggested that seasonal changes in SB in childhood are not well-established [13]. We have identified only two previous studies that examined seasonal changes in both objectively measured PA and SB in children [14, 15], and the results of these two studies were not consistent. Seasonality in PA and SB can be affected by many factors, including climate, the school education system, and the evaluation methodology of PA and SB. Moreover, it remains unclear to what extent objectively measured habitual PA and SB changes longitudinally between the school year and summer vacation, and whether any changes are modified by gender in children. Our previous study [16] showed that Japanese preschool children spend more non-ambulatory activity than ambulatory activity during moderate intensity activity. The present study sought to examine longitudinal changes in habitual PA and SB measured objectively, in the school year and summer vacation in primary school aged Japanese children. Moreover, the determinants/moderators of objectively measured changes in SB and PA were examined (the influence of gender, sports participation, home environment and psychological aspects were considered).

## Methods

Our convenience sample included 209 Japanese primary children from 4 public primary schools in urban areas in Tokyo and Kyoto. Participants were invited to participate by leaflets, such as a newsletter, at their school. Informed consent was obtained from all participants and their parents, and the Ethical Committee of J. F. Oberlin University approved the study protocol (No. 10007). Baseline data of anthropometric measurements, SB and PA were collected in May 2011 during the school year. The average temperatures in the school period and summer vacation were 19.3 (standard deviation (SD) 2.4) degrees and 27.3 (SD 2.5) degrees, the maximum temperatures were 24.5 (SD 3.8) degrees and 31.6 (SD 3.3) degrees, and the average humidities were 59.9 (SD 12.0) % and 68.7 (SD 6.3) %. All temperature and humidity data in summer vacation were higher than those of the school term in spring [4].

### Objective measurement of sedentary behavior and physical activity

SB and PA in free-living conditions were evaluated with a triaxial accelerometer (Active style Pro HJA-350IT, Omron Healthcare, Kyoto), 74 × 46 × 34 mm and 60 g including batteries. Participants wore the accelerometer on the left side of the waist for both the school year and the summer vacation measurements. The device is described in detail elsewhere [17]. In brief, the synthetic acceleration of three axes using signals before and after high-pass filtering was calculated, and we obtained the ratio of the unfiltered to filtered synthetic acceleration. The average of the absolute value of the filtered acceleration from 10 s epochs was used to estimate PA intensity. Our previous study reported the algorithm for the classification of non-ambulatory activities such as playing games, throwing a ball, cleaning and clearing away and ambulatory activities such as walking and running by the unfiltered/filtered acceleration ratio [17]. Discrimination with the ratio provided the highest rate of correct discrimination, 99.8 % when the value of the ratio was 1.12. The percentage of correct discrimination with the ratio used by the Active style Pro (1.16) was comparable (98.7 %). Moreover, strong linear relationships were found for both non-ambulatory (metabolic equivalents (METs) = 0.0136 synthetic acceleration +1.220, R^2^ = 0.772) and ambulatory (METs = 0.0056 synthetic acceleration +0.944, R^2^ = 0.880) activities, except for climbing up and down. In fact, our previous study showed that non-ambulatory time as measured by triaxial accelerometry was much longer than ambulatory time during medium-intensity PA in free-living Japanese preschool children [16]. Our previous studies of adults showed that the relative contributions of non-ambulatory MVPA time and ambulatory MVPA time as measured by the Active style Pro depended on sex, age and occupation [18, 19]. Step counts were also measured, because they are used widely in many studies and investigations to objectively evaluate PA. When the Active style Pro is used for the evaluation of SB and PA in primary school children, the values of METs are overestimated [17], because the predictive equations were established for adults. Therefore, we used the following conversion equations for primary school children obtained from the results of Hikihara et al. [17]:

$$ \mathrm{The}\ \mathrm{ambulatory}\ \mathrm{activities}:\ 0.6237\times \mathrm{the}\ \mathrm{value}\ \mathrm{of}\ \mathrm{MET}\ \mathrm{b}\mathrm{y}\ \mathrm{the}\ \mathrm{Active}\ \mathrm{style}\ \mathrm{Pro} + 0.2411 $$

$$ \mathrm{The}\ \mathrm{n}\mathrm{o}\mathrm{n}\hbox{-} \mathrm{ambulatory}\ \mathrm{activities}:\ 0.6145\times \mathrm{the}\ \mathrm{value}\ \mathrm{o}\mathrm{f}\ \mathrm{MET}\ \mathrm{b}\mathrm{y}\ \mathrm{the}\ \mathrm{Active}\ \mathrm{style}\ \mathrm{Pro} + 0.5573 $$

The duration of ambulatory or non-ambulatory activity in each intensity were calculated. Moreover, total PA in each intensity was obtained as a sum of ambulatory time and non-ambulatory time.

SB and PA were monitored continuously for 7 days or more. In the summer vacation, some participants couldn’t return the device on the same day. Therefore, participants were requested to remove the device after 7 days or more from the beginning of the measurement. Participants were requested to wear the device at all times, except under special circumstances, such as dressing, bathing and swimming. Non-wear time was defined as periods with over 1 h of consecutive zero counts. In fact, many participants wore the accelerometer during sleep. Because sleep and sedentary time cannot be discriminated, we analyzed data collected between 7:00 and 21:00 to exclude sleep time. We included days with more than 10 h (600 min) of wearing time per day. The accelerometry data reduction criteria used in the present study were similar to those in other papers [20]. Penpraze et al. [21] indicated that the reliability of PA monitoring was nearly the same from 3 to 10 days in young children. Cliff et al. [22] suggested at least 3 days of monitoring in young children was required for reliable measures. Participants with data from at least 2 weekdays and at least 1 weekend day in the school years and at least 3 days in the summer vacation were included in the analysis. Participants attended classes at their school on weekdays in the school year and lived freely on weekend. On the other hand, they went to their school on neither weekdays nor weekend days in the summer vacation. Thus, during the summer vacation, the minimal number of days was set at 3 days without differentiation between weekend and weekdays.

### Potential determinants/moderators of changes in sedentary time and physical activity

The present study considered potential moderators of seasonal changes using a socio-ecological model as recommended [23–25]. Some items like demographic and biological, psychological and behavioral domains have been considered in public health surveillance with physical fitness in children and youth in Japan, in surveys carried out by the Ministry of Education, Culture, Sports, Science and Technology, by the Japan Sports Agency, by a national survey of Japanese Society of School Health, and finally by a survey carried out by Sasagawa sports foundation [1, 26, 27]. We included as potential moderators those variables which were considered important in Japan and which can be measured using standard questionnaires used widely in Japanese public health surveillance.

The variables studied for each domain were:

- a demographic and biological domain: gender; age.
- a psychological domain: body image, perception of sports, health and activity.
- a behavioral domain: attendance at sports clubs.
- a physical environmental domain: television set in children’s bedroom.

The data were collected from participants who answered with their parents. Only the psychological domain was collected by personal interview for children or questionnaire for their parents, respectively.

### Anthropometric measurements

We measured participants’ body height and body weight to the nearest 0.1 cm and 0.1 kg, respectively. Height and body weight was measured without shoes, but with clothing. Net body weight was calculated as the weight of clothing subtracted from the measured body weight. We measured the anthropometric measurements once at each season according to the method described by School Health Survey of the Ministry of Education, Culture, Sports, Science and Technology [28]. We calculated body mass index (BMI) as weight in kilograms divided by height in meters squared. Weight status was classified as normal weight, overweight/obese, or thin using Japanese cut-offs for weight status that were established based on national reference data for Japanese children [28]. Relative weight was calculated as follows:

Relative weight = [measured body weight (kg) – standard weight for gender, age, and height (kg)]/ standard weight for gender, age, and height (kg) × 100 (%)

※ standard weight for gender, age, and height (kg) = a × measured height (cm) – b

a and b are gender- and age-specific.

The cut-offs of weight status are as follows: Overweight/ Obesity combined: ≥ + 120 %, Normal weight: −120 + 120 % and Thinness: ≤ − 120 %.

### Analyses

The time spent at SB and each PA intensity per day was calculated by METs: average number of weekday and weekend minutes spent in SB (METs ≤ 1.5), LPA (1.5 < METs < 3.0), MVPA (3.0 ≤ METs), moderate PA (MPA) (3.0 ≤ METs <5.9) were calculated for each individual, and then average weekly values were calculated. For the data in the school year, average values were calculated by weighting for 5 weekdays and 2 weekend days (Weighted data = (average for weekdays × 5) + (average for weekend days × 2) / 7). The PA assessed by the accelerometer is presented as: (1) ambulatory activity or non-ambulatory activity in each intensity category (LPA, MVPA and MPA); and (2) number of steps registered per day. Values of SB and PA were adjusted by baseline and follow-up wear time, respectively.

The initial sample comprised 356 participants. Due to missing data (no consent to take part/unable to trace for follow-up measures [*n* = 46], no accelerometer data at baseline or follow-up [*n* = 94], no height/weight data at follow-up [*n* = 7]), our longitudinal sample comprised data from 209 children. A few questions weren’t answered completely by children or parents. Therefore, there were missing data in the analysis of the determinants/moderators of objectively measured changes in SB and PA. Numbers of each analysis were described in each Table. There was no significant difference between the relative weight at baseline of the participants and children who dropped out (boys: *p* = 0.101, girls: *p* = 0.391). The follow-up data were collected at the end of July or middle of August during the summer vacation (mean interval, 64 (SD 10) days).

A paired sample t-test was used to compare baseline and follow-up measurements, for each gender. The associations between change in SB or PA and determinants variables at baseline were analyzed by analysis of covariance (ANCOVA) adjusted for school, follow-up period, age, SB or PA at baseline. Moreover, when each first analysis was signifıcant, in the fınal stage of the analysis, SB or PA variables were also adjusted in the same model. When the number of each answer was below 10 participants, the category was added to the next other category. Results are shown as means ± SD. Statistical analysis was performed with IBM SPSS statistics 20.0 for Windows (IBM Co., Tokyo, Japan). *P* < 0.05 was considered significant.

## Results

### Characteristics of study participants

The characteristics of study participants are presented in Table 1. Average age for boys and girls was 8.9 (SD 1.8) years old and 9.1 (SD 1.8) years old at baseline, respectively. Five percent of boys and 6 percent of girls were overweight/obese. The duration of accelerometry was much greater than the minimum criteria specified (at least 3 days and 10 h), with an average of 7.4 days and 13.3 h for boys, and 8.1 days and 12.8 h for girls at baseline and 7.2 days and 13.4 h for boys, and 8.8 days and 12.8 h for girls at follow-up, respectively. The percentage of the sample which had 2 weekdays and 1 weekend day data was 95 % for boys and 93 % for girls at follow-up, respectively. The results of psychological, behavioral and physical environmental domains are shown in Table 1.

**Table 1:** Physical characteristics and determinants/moderators at baseline for study participants

	Boys (*n* = 98)		Girls (*n* = 111)	
	Average ± SD		Average ± SD	
Height (cm)	130.9 ± 11.1		132.7 ± 12.1	
Body weight (kg)	29.5 ± 8.4		29.2 ± 7.7	
Body mass index (kg/m^2^)	16.9 ± 2.4		16.3 ± 2.1	
Weight status (overweight and obese: %)	5.1		6.3	
Participation in sports except for physical education (n)	95		107	
1. yes	66		77	
2. no	29		30	
Duration (min/week) (boys *n* = 58, girls *n* = 68)	192.2 ± 156.0		152.5 ± 157.6	
Duration (min/time) (boys *n* = 58, girls *n* = 68)	80.0 ± 30.8		79.9 ± 37.7	
Frequency (time/week) (boys *n* = 58, girls *n* = 68)	2.3 ± 1.5		1.8 ± 1.3	
Bedroom television ownership (*n*)	96		109	
1. yes	18		28	
2. no	78		81	
	Children	Parents	Children	Parents
Are you (Is your child) active? (n)	98	96	109	109
1. no	9	13	10	13
2. neither	34	36	42	43
3. yes	55	47	57	53
Do you (Does your child) like sports or exercise? (n)	97	97	111	109
1. considerably dislike	0	2	0	1
2. slightly dislike	2	12	8	11
3. slightly like	22	26	21	34
4. considerably like	73	57	82	63
Are you (Is your child) good at sports or exercise? (*n*)	98	97	111	109
1. considerably lower skilled	1	3	2	5
2. slightly lower skilled	9	31	21	34
3. slightly highly skilled	40	32	34	40
4. considerably highly skill	48	31	54	30
Are you (Is your child) healthy? (*n*)	98	97	111	109
1. not healthy	0	1	2	5
2. somewhat healthy	4	0	21	34
3. healthy	45	53	34	40
4. very healthy	48	43	54	30
How would you describe your (your child’s) body shape?	98	97	111	109
1. considerably thin	1	1	5	3
2. slightly thin	8	31	14	28
3. maintain the present body	72	47	83	66
4. over weight	15	17	8	6
5. Obese	2	1	1	3

Japanese boys and girls participated in this research in 2011, Abbreviations:﻿ *SD* s﻿tandard deviatio﻿n

Time spent at different activity intensity levels for ambulatory and non-ambulatory activity and total time, and step counts are shown in Table 2. Daily SB significantly increased from baseline to follow-up for boys (from a mean of 341 to 354 min) and girls (from a mean of 357  to 371 min). Ambulatory and total time in LPA, MVPA and MPA, non-ambulatory in LPA and step counts for boys significantly decreased from baseline to follow-up (e.g., from 76 to 65 min for total MVPA). Ambulatory, non-ambulatory and total time in LPA, MVPA and MPA, and step counts for girls significantly decreased from baseline to follow-up (e.g., from 61 minutes to 51 min for total MVPA).

**Table 2:** Gender differences in seasonality in sedentary behavior and physical activity for study participants

	Boys (*n* = 98)						Girls (*n* = 111)					
	Baseline	Follow-up	t	95 % CI		*P*-value	Baseline	Follow-up	t	95 % CI		*P*-value
	Average SD	Average SD					Average SD	Average SD				
Sedentary behavior (min/day)	340.6 ± 69.4	354.0 ± 73.8	−2.5	−24.2	−2.7	0.015	356.5 ± 60.6	370.6 ± 71.5	−2.7	−24.2	−3.8	0.008
LPA (min/day)												
Ambulatory	107.5 ± 20.4	91.4 ± 27.5	7.0	11.5	20.7	<0.001	104.1 ± 17.4	88.6 ± 23.0	8.7	12.0	19.0	<0.001
Non-ambulatory	262.6 ± 48.9	246.5 ± 56.4	3.6	7.1	25.1	0.001	271.6 ± 45.4	244.4 ± 49.9	8.5	20.9	33.5	<0.001
Total time	370.1 ± 59.2	337.8 ± 69.4	5.9	21.5	43.1	<0.001	375.7 ± 50.8	333.0 ± 60.2	10.5	34.6	50.7	<0.001
MVPA (min/day)												
Ambulatory	46.5 ± 15.0	35.5 ± 19.8	7.1	7.9	14.0	<0.001	33.0 ± 9.9	25.4 ± 11.4	8.6	5.9	9.4	<0.001
Non-ambulatory	29.4 ± 9.6	29.8 ± 11.2	−0.5	−1.9	1.1	0.593	27.9 ± 7.6	25.3 ± 7.7	4.7	1.5	3.7	<0.001
Total time	75.9 ± 21.5	65.3 ± 26.6	5.5	6.8	14.4	<0.001	60.9 ± 15.4	50.6 ± 16.4	8.5	7.9	12.7	<0.001
MPA (min/day)												
Ambulatory	40.5 ± 12.9	32.2 ± 17.2	5.6	11.0	−9.6	<0.001	29.4 ± 8.8	23.5 ± 10.5	7.4	4.3	7.5	<0.001
Non-ambulatory	27.4 ± 8.9	28.4 ± 10.7	−2.5	0.4	2.1	0.154	26.6 ± 7.4	24.3 ± 7.5	4.3	1.2	3.3	<0.001
Total time	68.0 ± 18.6	60.6 ± 23.2	4.1	10.8	−8.2	<0.001	55.9 ± 14.0	47.8 ± 15.2	7.4	5.9	10.3	<0.001
Step count (steps/day)	12152 ± 2804	9860 ± 3863	7	1663	2921	<0.001	10408 ± 1808	8583 ± 2484	9	1410	2240	<0.001

*Abbreviations*: *LPA* light physical activity, *MVPA* moderte-to-vigorous physical activity, *MPA* moderate physical activity, *SD* standard deviat﻿io﻿n, *CI* confidence interval

### Determinants/moderators of changes in sedentary behavior and physical activity

The decrease in non-ambulatory MVPA was significantly lower for boys who participated in sports, both for the school periods and summer vacation, than those who did not (Table 3). The increase in SB, and decrease in ambulatory LPA and step counts were significantly lower for girls who participated in sports than those who did not (Table 3). The change in SB for boys was significantly associated with bedroom television (TV) ownership (Table 4), with significantly more adverse change in SB in those who had a TV in the bedroom.

**Table 3:** Associations between changes in sedentary behavior or physical activity and participation in sports

Dependent variables	Participation in sports both for the school periods and summer vacation^a^	Boys (No: *n* = 10, Yes: *n* = 58)				Girls (No: *n* = 12, Yes: *n* = 72)			
		Estimated mean	SE	B	*P*-value	Estimated mean	SE	B	*P*-value
Δsedentary behavior (min/day)	No	30.0	19.3	28.8	0.138	47.9	15.9	36.4	0.032
	Yes	1.2	9.6	0.0		11.6	6.8	0.0	
Δsedentary behavior (min/day) ^b^	No	29.0	19.5	29.5	0.131	48.2	16.1	36.8	0.033
	Yes	-0.5	9.9	0.0		11.3	7.0	0.0	
ΔLPA (min/day) Ambulatory	No	−25.9	7.6	−12.2	0.110	−26.4	5.0	−14.0	0.010
	Yes	−13.7	3.8	0.0		−12.4	2.2	0.0	
Ambulatory ^b^	No	−26.0	7.7	−12.1	0.117	−26.5	5.1	−14.3	0.009
	Yes	−13.9	4.0	0.0		−12.2	2.2	0.0	
Non-ambulatory	No	−39.6	15.5	−17.8	0.253	−35.8	10.2	−9.6	0.374
	Yes	−21.8	7.5	0.0		−26.3	4.4	0.0	
Non-ambulatory ^b^	No	−38.1	15.6	−18.1	0.246	−35.8	10.2	−9.6	0.376
	Yes	−20.0	7.7	0.0		−26.3	4.4	0.0	
Total	No	−65.4	18.6	−30.5	0.104	−62.4	12.4	−24.0	0.070
	Yes	−34.9	9.1	0.0		−38.5	5.3	0.0	
Total ^b^	No	−64.7	18.8	−30.6	0.105	−62.4	12.5	−24.0	0.071
	Yes	−34.1	9.3	0.0		−38.4	5.3	0.0	
ΔMVPA (min/day) Ambulatory	No	−8.5	4.7	−4.4	0.346	−9.2	2.4	−2.6	0.297
	Yes	−4.1	2.3	0.0		−6.6	1.0	0.0	
Ambulatory ^b^	No	−8.8	4.7	−4.4	0.355	−9.4	2.3	−2.9	0.242
	Yes	−4.5	2.4	0.0		−6.5	1.0	0.0	
Non-ambulatory	No	−5.4	2.5	−5.1	0.041	−3.9	1.6	−1.9	0.261
	Yes	−0.2	1.3	0.0		−2.1	0.7	0.0	
Non-ambulatory ^b^	No	−5.4	2.5	−5.0	0.047	−4.1	1.6	−2.1	0.206
	Yes	−0.4	1.3	0.0		−2.0	0.7	0.0	
Total	No	−13.6	6.2	−9.4	0.130	−13.2	3.2	−4.5	0.188
	Yes	−4.2	3.2	0.0		−8.7	1.4	0.0	
Total ^b^	No	−13.9	6.2	−9.3	0.133	−13.7	3.1	−5.3	0.117
	Yes	−4.6	3.2	0.0		−8.5	1.4	0.0	
ΔMPA (min/day) Ambulatory	No	−6.6	4.0	−3.7	0.354	−6.8	2.2	−1.9	0.413
	Yes	−2.9	2.0	0.0		−4.9	1.0	0.0	
Ambulatory ^b^	No	−6.8	4.1	−3.7	0.360	−7.0	2.2	−2.2	0.349
	Yes	−3.1	2.0	0.0		−4.9	0.9	0.0	
Non-ambulatory	No	−4.8	2.4	−4.6	0.057	−3.3	1.5	−1.3	0.402
	Yes	−0.2	1.2	0.0		−2.0	0.6	0.0	
Non-ambulatory ^b^	No	−4.8	2.4	−4.5	0.064	−3.5	1.5	−1.6	0.331
	Yes	−0.3	1.2	0.0		−2.0	0.6	0.0	
Total	No	−11.7	5.4	−8.6	0.113	−10.0	2.9	−3.3	0.293
	Yes	−3.1	2.7	0.0		−6.7	1.3	0.0	
Total ^b^	No	−11.9	5.4	−8.5	0.116	−10.6	2.9	−4.0	0.190
	Yes	−3.3	2.8	0.0		−6.5	1.2	0.0	
ΔStep count (steps/day)	No	−2912	1010	−1666	0.100	−2885	592	−1377	0.029
	Yes	−1246	492	0		−1508	257	0	

*Abbreviations*: *LPA* light physical activity, *MVPA* moderate-to-vigorous physical activity, *MPA* moderate physical activity, *SE* standard error, Δ change, Δvariables were calculated as follow-up values minus baseline values

^a^ except for physical education in the school period, adjusted for school, follow-up periods, age and sedentary behavior or physical activity at baseline

^b^ adjusted for sedentary behavior or moderate-to-vigorous physical activity at baseline

**Table 4:** Associations between changes in sedentary behavior or physical activity and a television in the bedroom

Dependent variables	Ownership of television at the participant’s bedroom	Boys (Yes: *n* = 18, No: *n* = 78)				Girls (Yes: *n* = 28, No: *n* = 81)			
		Estimated mean	SE	B	*P*-value	Estimated mean	SE	B	*P*-value
Δsedentary behavior (min/day)	Yes	26.5	11.2	25.9	0.036	24.7	10.3	14.6	0.204
	No	0.6	6.2	0.0		10.1	6.0	0.0	
Δsedentary behavior (min/day) ^a^	Yes	26.5	11.0	28.1	0.022	24.7	10.3	15.0	0.196
	No	−1.7	6.3	0.0		9.8	6.1	0.0	
ΔLPA (min/day) Ambulatory	Yes	−17.2	5.0	−3.9	0.477	−16.5	3.5	−2.5	0.510
	No	−13.3	2.8	0.0		−14.0	2.0	0.0	
Ambulatory ^a^	Yes	−17.2	5.0	−3.5	0.525	−16.6	3.5	−2.6	0.507
	No	−13.7	2.8	0.0		−14.0	2.0	0.0	
Non-ambulatory	Yes	−26.1	9.7	−12.0	0.259	−32.5	6.5	−6.1	0.398
	No	−14.2	5.3	0.0		−26.3	3.8	0.0	
Non-ambulatory ^a^	Yes	−25.9	9.8	−12.2	0.253	−32.1	6.6	−5.8	0.429
	No	−13.7	5.4	0.0		−26.3	3.8	0.0	
Total	Yes	−43.5	11.2	−15.8	0.200	−48.8	8.2	−8.6	0.346
	No	−27.8	6.1	0.0		−40.2	4.8	0.0	
Total ^a^	Yes	−43.8	11.3	−15.6	0.206	−48.7	8.3	−8.5	0.359
	No	−28.1	6.2	0.0		−40.2	4.8	0.0	
ΔMVPA (min/day) Ambulatory	Yes	−12.7	3.5	−6.2	0.109	−7.7	1.5	−1.0	0.531
	No	−6.5	1.9	0.0		−6.6	0.9	0.0	
Ambulatory ^a^	Yes	−12.8	3.5	−5.9	0.121	−7.9	1.5	−1.3	0.430
	No	−6.9	1.9	0.0		−6.6	0.9	0.0	
Non-ambulatory	Yes	0.5	1.6	−0.2	0.892	−3.7	1.0	−1.8	0.121
	No	0.7	0.9	0.0		−1.9	0.6	0.0	
Non-ambulatory ^a^	Yes	0.5	1.6	−0.2	0.912	−3.7	1.0	−1.9	0.102
	No	0.7	0.9	0.0		−1.9	0.6	0.0	
Total	Yes	−12.3	4.2	−6.6	0.159	−11.4	2.0	−2.8	0.215
	No	−5.7	2.4	0.0		−8.6	1.2	0.0	
Total ^a^	Yes	−12.4	4.2	−6.5	0.160	−11.7	2.0	−3.3	0.139
	No	−5.9	2.4	0.0		−8.4	1.2	0.0	
ΔMPA (min/day) Ambulatory	Yes	−10.7	3.1	−6.3	0.063	−5.9	1.4	−0.8	0.600
	No	−4.4	1.7	0.0		−5.1	0.8	0.0	
Ambulatory ^a^	Yes	−10.8	3.0	−6.1	0.069	−6.0	1.4	−1.0	0.510
	No	−4.6	1.7	0.0		−5.0	0.8	0.0	
Non-ambulatory	Yes	1.2	1.5	0.1	0.960	−3.3	1.0	−1.6	0.133
	No	1.1	0.9	0.0		−1.7	0.6	0.0	
Non-ambulatory ^a^	Yes	1.2	1.6	0.1	0.956	−3.4	1.0	−1.7	0.120
	No	1.1	0.9	0.0		−1.7	0.6	0.0	
Total	Yes	−9.8	3.7	−6.4	0.118	−9.3	1.9	−2.8	0.180
	No	−3.4	2.1	0.0		−6.6	1.1	0.0	
Total ^a^	Yes	−9.9	3.7	−6.4	0.117	−9.5	1.8	−3.1	0.127
	No	−3.5	2.1	0.0		−6.4	1.1	0.0	
ΔStep count (steps/day)	Yes	−2337	701	−758	0.329	−1820	385	−197	0.646
	No	−1580	396	0		−1623	228	0	

*Abbreviations*: *LPA* light physical activity, *MVPA* moderate-to-vigorous physical activity, *MPA* moderate physical activity, *SE* standard error, Δ change, Δvariables were calculated as follow-up values minus baseline values, adjusted for school, follow-up periods, age and sedentary behavior or m﻿oderate-to-vigorous physical activity at baseline

The perception of sports and body image by boys, health perception by boys’ parents, activity and sports perceptions by girls, and the sports perceptions and body image by girls’ parents were associated with change in SB and PAs, respectively (see attached Additional files 1 and 2: Table S1a, Table S1b). The decrease in PA was significantly lower in boys with positive perceptions of sports than those who had negative perceptions. The decrease in PA was significantly lower in boys with negative perceptions of their child’s health than those who had positive perceptions by their parents. The decrease in PA in non-ambulatory activity was significantly higher in boys with overweight or obese perception than those who wanted to maintain the present body. The decrease in MPA was significantly lower in girls or their parents with positive perceptions of sports than those who had negative perceptions. The decrease in MPA in ambulatory activity was significantly lower in girls with overweight or obese perception than those who wanted to maintain the present body by their parents.

## Discussion

This study examined the longitudinal changes of objectively evaluated SB and PA, between the school year and summer vacation in Japanese primary school children. To our knowledge, no previous study has addressed changes in objectively-evaluated sedentary time and physically activity with discrimination between ambulatory and non-ambulatory PA in elementary school children at the school year and summer vacation. As we hypothesized, SB increased and PA decreased significantly in the summer vacation in both genders. Adverse changes in the summer vacation were moderated by membership of sports clubs, not having a TV in the bedroom, positive perceptions of sports for boys or positive perceptions of sports and activity for girls and their parents and negative perception of boy’s health for their parents. In detail, there were significant decreases in ambulatory and total LPA, MVPA, MPA and step counts in the summer vacation in both genders, while non-ambulatory MVPA and MPA were significantly lower in the summer vacation just in girls.

A previous review [13] described the influence of season on accelerometer-determined measures of SB and PA in children. Significant seasonal variation in PA was reported in all UK studies, being highest in summer and lowest in winter. In non-UK studies (other European countries, USA and New Zealand) significant seasonal variation in PA was not found, and findings were inconclusive for SB [13]. Recently, Lewis et al. reported that daily maximum temperature was significantly associated with MVPA and SB time in Australia and Canada. MVPA and SB time appear to be optimal when the maximum temperature ranges between 20 and 25 °C in both countries [5]. In the present study, the maximum temperatures were 24.5 (SD 3.8) degree in the school year and 31.6 (SD 3.3) degree in summer vacation. Moreover, the average humidities in the summer vacation (68.7 (SD 6.3) %) were also higher than in the school year (59.9 (SD 12.0) %) [4]. Thus, weather characteristics might affect seasonal change of PA and SB in Japan, as in Australia and Canada. Moreover, another review [29] identified only two previous studies that examined change in total energy expenditure or objectively measured SB and PA between the school year and summer vacation in children. Zinkel et al. [14] demonstrated a seasonal pattern in total energy expenditure by doubly labeled water method in a cross-sectional study of 6-to-13 year old children in the greater Washington DC area, total energy expenditure was higher during the school year. However, after statistically controlling for fat free mass, total energy expenditure was no longer significantly seasonal. McCue et al. [15] reported that SB increased and LPA and MPA declined (but not MVPA or vigorous PA) by accelerometry in longitudinal designed study for 9-to-11 year old Minnesota children. The present study findings are similar to the results of McCue et al.’s study on the change in SB, LPA and MPA. However, MVPA changes in the present study were not similar: one of the reasons might be the difference in weather. Hot and humid weather during the summer vacation both in Tokyo and Kyoto may have kept participants from going outside, resulting in decreased PA and increased SB. In addition, accelerometry used in the present study and that in the previous studies were quite different. The Active style Pro can accurately discriminate ambulatory and non-ambulatory PA, while ActiGraph with the cut point method tends to underestimate non-ambulatory PA [17]. Another possible reason for differences between studies is that McCue et al. [15] studied a small sample (19 boys and 11 girls).

There appears to be even less comparable literature on the determinants or moderators of seasonal changes in PA and SB in children. Rowlands et al. [30] considered the influence of gender on seasonal variation in PA in a longitudinal study of 64 nine to 11 year old UK children measured in summer and winter. Seasonal differences in activity level were largest for weekday activity in boys and only present for weekend activity in girls, where activity levels were higher in the summer than the winter. On the other hand, the impact of season on ambulatory and total LPA, MVPA and MPA, non-ambulatory LPA, SB and step counts were comparable between genders in the present study. Only girls spent less time in non-ambulatory MVPA and MPA in summer vacation than the school year. The reason for the reduction in non-ambulatory PA during the summer vacation in the present study is not clear. However, for Japanese adult women, habitual time spent in non-ambulatory MVPA is longer than that of men [19]. Evidence from our study supports the suggestion that not only ambulatory activity but also non-ambulatory activity is an important factor in evaluating PA in girls. These data may be particularly important for providing insight into improving gender-related disparities in PA in children.

The present study suggested a possible gender difference in the determinants/moderators of seasonal changes in PA and SB. For boys, participation in sports, not having a television set in the boy’s bedroom, positive perceptions of sports by themselves and the negative perceptions of child’s health by their parents might be better targets for intervention for boy’s SB and non-ambulatory and ambulatory PAs. In the case of girls, participation in sports, the positive perceptions of or highly perceived competence in sports or activity by themselves or their parents and the negative perceptions of child’s body by their parents might be important for girls’ SB and PA changes in the summer vacation. Recent reviews reported that sport participants have more PA than those who do not participate and higher engagement with sport and PA can lead to improvements in self-esteem, at least in the short term [31, 32]. Moreover, another review showed that having a television set in the bedroom was positively associated with TV viewing time [33].

There were several limitations to the current study. The accelerometer used in the present study has been validated and has been widely used to evaluate PA in Japan, but may not accurately assess all types of PA, such as swimming, cycling and bathing. Nixon et al. [34] reported that objectively measured seasonal differences in sleep duration in 7 year old children with actigraphy found substantially less sleep in the summer than during the school year. Sleep duration varies considerably among individuals. The duration is affected by day of the week, season, and having younger siblings. The present study might be missing shifts in wake-sleep times that may be seasonal and the differences in wake and sleep time between different children. Future studies should consider sleep times where possible, though this is presents practical problems for researchers. For example, Dayyat et al. [35] reported that the description of a child’s sleep by the parent does not result in a correct estimate of sleep onset or duration. Therefore, the present study stipulated a set sleep time for all participants. The vast majority of children in the present study were not obese. As a consequence, it may not be appropriate to extend our results to more obese populations. The potential moderators of seasonal changes were limited to those readily available, and other potential moderators (e.g. socio-economic status) might have been important but were not measured and not readily available. Finally, while the present study identified seasonal changes, the precise reasons why these changes occurred (weather, differences in behavior between school days and school holidays) could not be confirmed. Nonetheless, the present study had a number of strengths. The longitudinal design and objective measures of PA and SB were strengths. The study had a larger sample size than most previous longitudinal studies of seasonality in children. Finally, the relatively short period between baseline and follow up measures in the present study would have minimized potential age/maturation-related differences in the behaviors measured, so that any changes could be interpreted as seasonal rather than maturational. Future studies should prospectively examine the change in patterns of SB to obtain more evidence on this important issue.

## Conclusions

These present study suggests that Japanese primary school children of both genders have higher SB and lower LPA and ambulatory MVPA during the summer vacation. The results emphasize the need to take summer vacation into account when developing PA or SB interventions for primary school children in Japan. This study also suggests some factors that may moderate seasonal changes in PA and SB in Japanese children: sports participation may mitigate the adverse changes for boys and girls; television in the bedroom may exacerbate the seasonal change.

## Additional files

Additional file 1: Table S1a. Associations between change of physical activity and children’s perception or their parents’ perception of sports, body image or health in boys (PDF 124 kb) Additional file 2: Table S1b. Associations between change of sedentary behavior or physical activity and children’s perception or their parents’ perception of sports or body image in girls (PDF 103 kb)

## Abbreviations

LPA: Light intensity activity
METs: Metabolic equivalents
MPA: Moderate physical activity
MVPA: Moderate-to-vigorous physical activity
PA: Physical activity
SB: Sedentary behavior
TV: Television
