# Supplementary material for: Seasonal changes in objectively measured sedentary behavior and physical activity in Japanese primary school children
Source: BMC Public Health. 2016 Sep 13;16(1):969. doi: 10.1186/s12889-016-3633-5 (PMC5020446; doi:10.1186/s12889-016-3633-5)
Supplement: Additional file 1: Table S1a. — Associations between change of physical activity and children’s perception or their parents’ perception of sports, body image or health in boys (PDF 124 kb) [file 12889_2016_3633_MOESM1_ESM.pdf]

Additional file Table1a Associations between changes in sedentary behavior or physical activity and potential determinants/moderators in boys

Dependent variables	Do you like sports or exercise?	Boys: 1: n=24, 2: n=73				Are you good at sports or exercise?	Boys: 1: n=10, 2: n=40, 3: n=48				How would you describe your body shape?	Boys: 1: n=9, 2: n=72, 3: n=17				Is your child healthy?	Boys: 1: n=54, 2: n=43			
		Estimated mean	SE	B	P-value		Estimated mean	SE	B	P-value		Estimated mean	SE	B	P-value		Estimated mean	SE	B	P-value
ΔSedentary behavior (min/day)	1. slightly dislike or like	9.1	11.1	-1.5	0.902	1. considerably and slightly lower skilled	-16.5	16.4	-27.1		1. thin	5.2	19.4	-25.5		1 not healthy and healthy	-0.2	7.8	-19.1	0.073
	2. considerably like	10.6	7.0	0.0		2. slightly highly skilled	13.5	8.8	2.9		2. maintain the present body	3.3	7.2	-27.4		2 very healthy	18.9	8.3	0.0	
						3. considerably highly skill	10.6	8.4	0.0		3. overweight or obese	30.7	16.1	0.0						
	*1. slightly dislike or like	8.4	11.1	0.1	0.996	*1. considerably and slightly lower skilled	-12.6	17.1	-21.5		*1. thin	2.9	19.3	-29.7		*1 not healthy and healthy	-1.7	7.9	-18.6	0.080
	*2. considerably like	8.3	7.2	0.0		*2. slightly highly skilled	11.7	9.1	2.8		*2. maintain the present body	0.7	7.4	-32.0		*2 very healthy	16.9	8.4	0.0	
						*3. considerably highly skill	8.9	8.7	0.0		*3. overweight or obese	32.6	16.1	0.0						
ΔLPA (min/day)																				
Ambulatory	1. slightly dislike or like	-20.9	4.4	-7.8	0.117	1. considerably and slightly lower skilled	-21.8	6.8	-9.2		1. thin	-17.5	8.1	1.9		1 not healthy and healthy	-9.8	3.1	10.6	0.016
	2. considerably like	-13.1	2.9	0.0		2. slightly highly skilled	-13.8	3.6	-1.3		2. maintain the present body	-12.6	3.0	6.7		2 very healthy	-20.4	3.5	0.0	
						3. considerably highly skill	-12.6	3.7	0.0		3. overweight or obese	-19.4	6.7	0.0						
	*1. slightly dislike or like	-22.0	4.4	-8.8	0.077	*1. considerably and slightly lower skilled	-24.7	6.9	-12.9		*1. thin	-17.7	8.1	0.6		*1 not healthy and healthy	-9.9	3.2	10.5	0.017
	*2. considerably like	-13.2	2.9	0.0		*2. slightly highly skilled	-14.6	3.6	-2.7		*2. maintain the present body	-13.3	3.1	5.1		*2 very healthy	-20.5	3.5	0.0	
						*3. considerably highly skill	-11.8	3.7	0.0		*3. overweight or obese	-18.3	6.8	0.0						
Non-ambulatory	1. slightly dislike or like	-5.6	9.1	15.7	0.123	1. considerably and slightly lower skilled	11.3	13.2	26.3	0.017 (1 VS 2)	1. thin	1.7	16.2	42.0		1 not healthy and healthy	-15.7	6.6	4.7	0.600
	2. considerably like	-21.3	5.7	0.0		2. slightly highly skilled	-30.1	7.1	-15.2		2. maintain the present body	-14.9	5.8	25.4		2 very healthy	-20.4	7.1	0.0	
						3. considerably highly skill	-14.9	6.8	0.0		3. overweight or obese	-40.3	13.5	0.0						
	*1. slightly dislike or like	-5.1	9.2	15.8	0.123	*1. considerably and slightly lower skilled	11.4	13.3	25.8	0.023 (1 VS 2)	*1. thin	2.3	16.3	43.0		*1 not healthy and healthy	-15.0	6.8	5.2	0.567
	*2. considerably like	-20.9	5.8	0.0		*2. slightly highly skilled	-28.9	7.4	-14.4		*2. maintain the present body	-14.2	6.0	26.5		*2 very healthy	-20.2	7.1	0.0	
						*3. considerably highly skill	-14.5	6.9	0.0		*3. overweight or obese	-40.7	13.5	0.0						
Total	1. slightly dislike or like	-27.0	10.9	7.9	0.512	1. considerably and slightly lower skilled	-10.3	15.8	18.2		1. thin	-15.9	18.9	43.8		1 not healthy and healthy	-25.7	7.6	15.6	0.140
	2. considerably like	-34.9	6.9	0.0		2. slightly highly skilled	-44.3	8.5	-15.8		2. maintain the present body	-28.0	6.9	31.8		2 very healthy	-41.3	8.3	0.0	
						3. considerably highly skill	-28.5	8.2	0.0		3. overweight or obese	-59.8	15.7	0.0						
	*1. slightly dislike or like	-27.3	11.0	7.7	0.528	*1. considerably and slightly lower skilled	-10.9	16.1	17.6		*1. thin	-16.2	19.1	43.5		*1 not healthy and healthy	-25.7	7.9	15.6	0.150
	*2. considerably like	-35.0	7.0	0.0		*2. slightly highly skilled	-44.5	8.6	-16.1		*2. maintain the present body	-28.2	7.0	31.5		*2 very healthy	-41.3	8.4	0.0	
						*3. considerably highly skill	-28.5	8.3	0.0		*3. overweight or obese	-59.6	15.8	0.0						
ΔMVPA (min/day)																				
Ambulatory	1. slightly dislike or like	-14.4	3.0	-8.4	0.014	1. considerably and slightly lower skilled	-11.1	4.8	-3.8		1. thin	-17.2	5.7	-11.3		1 not healthy and healthy	-5.7	2.2	4.7	0.118
	2. considerably like	-6.0	1.9	0.0		2. slightly highly skilled	-7.3	2.6	0.1		2. maintain the present body	-7.3	2.1	-1.4		2 very healthy	-10.5	2.4	0.0	
						3. considerably highly skill	-7.4	2.5	0.0		3. overweight or obese	-5.8	4.7	0.0						
	*1. slightly dislike or like	-15.5	3.0	-9.2	0.006	*1. considerably and slightly lower skilled	-13.6	4.9	-6.4		*1. thin	-17.0	5.6	-11.6		*1 not healthy and healthy	-6.2	2.2	4.5	0.136
	*2. considerably like	-6.3	1.9	0.0		*2. slightly highly skilled	-7.5	2.6	-0.3		*2. maintain the present body	-7.8	2.1	-2.5		*2 very healthy	-10.7	2.4	0.0	
						*3. considerably highly skill	-7.2	2.5	0.0		*3. overweight or obese	-5.3	4.7	0.0						
Non-ambulatory	1. slightly dislike or like	-0.4	1.5	-1.1	0.511	1. considerably and slightly lower skilled	-1.1	2.2	-2.8		1. thin	2.5	2.6	7.7		1 not healthy and healthy	0.0	1.1	-1.0	0.480
	2. considerably like	0.7	1.0	0.0		2. slightly highly skilled	-0.4	1.2	-2.1		2. maintain the present body	1.8	1.0	7.0	0.014 (2 VS 3)	2 very healthy	1.1	1.2	0.0	
						3. considerably highly skill	1.7	1.2	0.0		3. overweight or obese	-5.2	2.1	0.0						
	*1. slightly dislike or like	-0.4	1.5	-1.1	0.526	*1. considerably and slightly lower skilled	-1.5	2.2	-3.1		*1. thin	2.5	2.6	7.5		*1 not healthy and healthy	0.0	1.1	-1.0	0.483
	*2. considerably like	0.6	1.0	0.0		*2. slightly highly skilled	-0.4	1.2	-2.0		*2. maintain the present body	1.7	1.0	6.7	0.021 (2 VS 3)	*2 very healthy	1.0	1.2	0.0	
						*3. considerably highly skill	1.7	1.2	0.0		*3. overweight or obese	-5.0	2.2	0.0						
Total	1. slightly dislike or like	-14.9	3.7	-9.4	0.025	1. considerably and slightly lower skilled	-12.1	5.8	-6.3		1. thin	-14.5	7.0	-3.5		1 not healthy and healthy	-5.7	2.8	3.8	0.314
	2. considerably like	-5.5	2.4	0.0		2. slightly highly skilled	-7.5	3.2	-1.7		2. maintain the present body	-5.4	2.7	5.6		2 very healthy	-9.5	3.0	0.0	
						3. considerably highly skill	-5.8	3.1	0.0		3. overweight or obese	-11.0	5.8	0.0						
	*1. slightly dislike or like	-15.7	3.7	-10.3	0.013	*1. considerably and slightly lower skilled	-15.8	5.9	-10.5		*1. thin	-14.2	6.9	-3.6		*1 not healthy and healthy	-6.0	2.8	3.5	0.347
	*2. considerably like	-5.4	2.4	0.0		*2. slightly highly skilled	-7.2	3.2	-2.0		*2. maintain the present body	-5.8	2.6	4.8		*2 very healthy	-9.5	3.0	0.0	
						*3. considerably highly skill	-5.3	3.0	0.0		*3. overweight or obese	-10.6	5.8	0.0						
ΔMPA (min/day)																				
Ambulatory	1. slightly dislike or like	-11.2	2.7	-0.6	0.023	1. considerably and slightly lower skilled	-8.6	4.2	-3.2		1. thin	-14.2	5.0	-10.6		1 not healthy and healthy	-3.9	2.0	4.2	0.119
	2. considerably like	-4.3	1.7	0.0		2. slightly highly skilled	-5.1	2.3	0.3		2. maintain the present body	-5.3	1.9	-1.7		2 very healthy	-8.1	2.1	0.0	
						3. considerably highly skill	-5.4	2.2	0.0		3. overweight or obese	-3.6	4.2	0.0						
	*1. slightly dislike or like	-12.0	2.6	-7.6	0.012	*1. considerably and slightly lower skilled	-10.5	4.4	-5.3		*1. thin	-14.0	5.0	-10.7		*1 not healthy and healthy	-4.2	2.0	4.0	0.138
	*2. considerably like	-4.4	1.7	0.0		*2. slightly highly skilled	-5.3	2.3	-0.1		*2. maintain the present body	-5.7	1.9	-2.4		*2 very healthy	-8.2	2.1	0.0	
						*3. considerably highly skill	-5.3	2.2	0.0		*3. overweight or obese	-3.3	4.1	0.0						
Non-ambulatory	1. slightly dislike or like	0.5	1.5	0.6	0.696	1. considerably and slightly lower skilled	-0.5	2.2	-2.5		1. thin	2.9	2.5	7.8		1 not healthy and healthy	0.6	1.1	-0.9	0.522
	2. considerably like	1.1	0.9	0.0		2. slightly highly skilled	0.2	1.2	-1.8		2. maintain the present body	2.3	0.9	7.3	0.008 (2 VS 3)	2 very healthy	1.5	1.1	0.0	
						3. considerably highly skill	2.0	1.1	0.0		3. overweight or obese	-4.9	2.1	0.0						
	*1. slightly dislike or like	0.5	1.5	-0.6	0.715	*1. considerably and slightly lower skilled	-0.7	2.2	-2.7		*1. thin	2.9	2.5	7.8		*1 not healthy and healthy	0.5	1.1	-0.9	0.517
	*2. considerably like	1.1	0.9	0.0		*2. slightly highly skilled	0.2	1.2	-1.7		*2. maintain the present body	2.3	0.9	7.2	0.010 (2 VS 3)	*2 very healthy	1.5	1.1	0.0	
						*3. considerably highly skill	2.0	1.1	0.0		*3. overweight or obese	-4.9	2.1	0.0						
Total	1. slightly dislike or like	-10.9	3.4	-7.3	0.051	1. considerably and slightly lower skilled	-8.8	5.1	-5.2		1. thin	-11.4	6.2	-2.5		1 not healthy and healthy	-3.6	2.5	3.3	0.327
	2. considerably like	-3.6	2.2	0.0		2. slightly highly skilled	-5.1	2.9	-1.5		2. maintain the present body	-3.1	2.3	5.9		2 very healthy	-6.8	2.7	0.0	
						3. considerably highly skill	-3.6	2.7	0.0		3. overweight or obese	-8.9	5.1	0.0						
	*1. slightly dislike or like	-11.4	3.3	-7.9	0.033	*1. considerably and slightly lower skilled	-11.8	5.3	-8.6		*1. thin	-11.0	6.1	-2.4		*1 not healthy and healthy	-4.1	2.5	3.6	0.291
	*2. considerably like	-3.5	2.1	0.0		*2. slightly highly skilled	-4.8	2.8	-1.6		*2. maintain the present body	-3.3	2.3	5.3		*2 very healthy	-7.7	2.7	0.0	
						*3. considerably highly skill	-3.2	2.7	0.0		*3. overweight or obese	-8.6	5.1	0.0						
ΔStep count (steps/day)	1. slightly dislike or like	-2883	601	-1399	0.042	1. considerably and slightly lower skilled	-2664	953	-1089		1. thin	-2461	1143	-349		1 not healthy and healthy	-1201	440	1322	0.030
	2. considerably like	-1483	397	0		2. slightly highly skilled	-1661	513	-86		2. maintain the present body	-1592	425	521		2 very healthy	-2523	487	0	
						3. considerably highly skill	-1576	515	0		3. overweight or obese	-2113	946	0						

LPA: Light physical activity, MVPA: moderate-to-vigorous physical activity, MPA: moderate physical activity, SE: standard error, Δ: change, Δvariables were calculated as follow-up values minus baseline values, adjusted for school, follow-up periods, age and sedentary behavior or physical activity at baseline, *: adjusted for school, follow-up periods, age and sedentary behavior or moderate-to-vigorous physical activity at baseline.
