# Supplementary material for: Seasonal changes in objectively measured sedentary behavior and physical activity in Japanese primary school children
Source: BMC Public Health. 2016 Sep 13;16(1):969. doi: 10.1186/s12889-016-3633-5 (PMC5020446; doi:10.1186/s12889-016-3633-5)
Supplement: Additional file 2: Table S1b. — Associations between change of sedentary behavior or physical activity and children’s perception or their parents’ perception of sports or body image in girls (PDF 103 kb) [file 12889_2016_3633_MOESM2_ESM.pdf]

Additional file Table1b Associations between changes in sedentary behavior or physical activity and potential determinants/moderators in girls

Dependent variables	Are you active?	Girls: 1: n=10, 2: n=42, 3: n=57				Are you good at sports or exercise?	Girls: 1: n=23, 2: n=34, 3: n=54				Is your child good at sports or exercise?	Girls: 1: n=39, 2: n=40, 3: n=30				How would you describe your child's body shape?	Girls: 1: n=31, 2: n=66, 3: n=12			
		Estimated mean	SE	B	P-value		Estimated mean	SE	B	P-value		Estimated mean	SE	B	P-value		Estimated mean	SE	B	P-value
ΔSedentary behavior (min/day)	1. no	9.4	17.3	-15.0		1. considerably and slightly lower skilled	22.4	11.4	7.4		1. considerably and slightly lower skilled	19.0	8.9	12.4		1. thin	17.1	11.2	35.8	
	2. neither	-0.1	8.1	-24.6	0.021 (2 VS 3)	2. slightly highly skilled	4.8	9.4	-10.2		2. slightly highly skilled	13.8	8.3	7.2		2. maintain the present body	17.8	6.8	36.5	
	3. yes	24.5	7.5	0.0		3. considerably highly skill	15.0	7.8	0.0		3. considerably highly skill	6.6	9.6	0.0		3. overweight or obese	-18.7	20.7	0.0	
	*1. no	9.0	17.5	-15.9		*1. considerably and slightly lower skilled	22.4	11.4	7.4		*1. considerably and slightly lower skilled	19.1	8.9	13.5		*1. thin	17.2	11.2	36.3	
	*2. neither	0.0	8.1	-24.8	0.021 (2 VS 3)	*2. slightly highly skilled	4.8	9.5	-10.1		*2. slightly highly skilled	13.7	8.4	8.2		*2. maintain the present body	17.5	6.9	36.6	
	*3. yes	24.8	7.6	0.0		*3. considerably highly skill	15.0	8.0	0.0		*3. considerably highly skill	5.5	9.9	0.0		*3. overweight or obese	-19.1	20.8	0.0	
ΔLPA (min/day)																				
Ambulatory	1. no	-12.3	5.9	3.7		1. considerably and slightly lower skilled	-19.2	3.8	-5.7		1. considerably and slightly lower skilled	-18.6	2.9	-7.9		1. thin	-15.4	3.8	-3.0	
	2. neither	-13.9	2.8	2.1		2. slightly highly skilled	-13.4	3.2	0.1		2. slightly highly skilled	-14.0	2.7	-3.3		2. maintain the present body	-14.3	2.3	-2.0	
	3. yes	-16.0	2.5	0.0		3. considerably highly skill	-13.5	2.6	0.0		3. considerably highly skill	-10.8	3.1	0.0		3. overweight or obese	-12.4	7.2	0.0	
	*1. no	-12.2	5.9	3.7		*1. considerably and slightly lower skilled	-19.2	3.8	-5.7		*1. considerably and slightly lower skilled	-18.9	3.0	-8.3		*1. thin	-15.4	3.8	-3.0	
	*2. neither	-13.9	2.8	2.1		*2. slightly highly skilled	-13.4	3.2	0.1		*2. slightly highly skilled	-14.0	2.8	-3.3		*2. maintain the present body	-14.3	2.3	-2.0	
	*3. yes	-16.0	2.6	0.0		*3. considerably highly skill	-13.5	2.6	0.0		*3. considerably highly skill	-10.6	3.2	0.0		*3. overweight or obese	-12.4	7.3	0.0	
Non-ambulatory	1. no	-37.5	10.7	-10.3		1. considerably and slightly lower skilled	-30.6	7.0	-2.5		1. considerably and slightly lower skilled	-33.9	5.6	-4.1		1. thin	-30.8	7.2	-11.6	
	2. neither	-24.4	5.0	2.8		2. slightly highly skilled	-25.1	5.8	2.9		2. slightly highly skilled	-21.0	5.2	8.8		2. maintain the present body	-27.7	4.3	-8.5	
	3. yes	-27.2	4.6	0.0		3. considerably highly skill	-28.1	4.8	0.0		3. considerably highly skill	-29.8	5.9	0.0		3. overweight or obese	-19.2	13.4	0.0	
	*1. no	-38.4	10.9	-11.8		*1. considerably and slightly lower skilled	-30.9	7.0	-3.2		*1. considerably and slightly lower skilled	-34.0	5.6	-4.7		*1. thin	-31.0	7.2	-12.7	
	*2. neither	-24.5	5.0	2.1		*2. slightly highly skilled	-24.9	5.8	2.8		*2. slightly highly skilled	-20.8	5.2	8.5		*2. maintain the present body	-27.4	4.4	-9.1	
	*3. yes	-26.6	4.8	0.0		*3. considerably highly skill	-27.7	4.9	0.0		*3. considerably highly skill	-29.3	6.0	0.0		*3. overweight or obese	-18.3	13.5	0.0	
Total	1. no	-50.4	13.5	-7.4		1. considerably and slightly lower skilled	-49.8	8.8	-8.2		1. considerably and slightly lower skilled	-52.3	6.9	-11.9		1. thin	-46.3	9.0	-14.8	
	2. neither	-37.9	6.3	5.0		2. slightly highly skilled	-38.0	7.3	3.6		2. slightly highly skilled	-34.9	6.5	5.4		2. maintain the present body	-41.8	5.4	-10.3	
	3. yes	-43.0	5.8	0.0		3. considerably highly skill	-41.6	6.1	0.0		3. considerably highly skill	-40.4	7.5	0.0		3. overweight or obese	-31.4	16.5	0.0	
	*1. no	-50.4	13.6	-7.5		*1. considerably and slightly lower skilled	-50.0	8.9	-8.5		*1. considerably and slightly lower skilled	-52.9	7.0	-13.2		*1. thin	-46.3	9.0	-14.9	
	*2. neither	-38.0	6.4	5.0		*2. slightly highly skilled	-38.1	7.3	3.4		*2. slightly highly skilled	-34.6	6.6	5.0		*2. maintain the present body	-41.6	5.5	-10.2	
	*3. yes	-43.0	6.0	0.0		*3. considerably highly skill	-41.5	6.2	0.0		*3. considerably highly skill	-39.6	7.6	0.0		*3. overweight or obese	-31.4	16.6	0.0	
ΔMVPA (min/day)																				
Ambulatory	1. no	-6.9	2.9	0.5		1. considerably and slightly lower skilled	-10.5	1.8	-4.5	0.032 (1 VS 2)	1. considerably and slightly lower skilled	-6.9	1.3	-1.9		1. thin	-8.2	1.6	-6.8	
	2. neither	-6.4	1.3	1.1		2. slightly highly skilled	-5.7	1.5	0.4		2. slightly highly skilled	-8.2	1.2	-3.2		2. maintain the present body	-7.3	1.0	-5.9	
	3. yes	-7.4	1.2	0.0		3. considerably highly skill	-6.1	1.2	0.0	0.037 (1 VS 3)	3. considerably highly skill	-5.0	1.4	0.0		3. overweight or obese	-1.4	3.0	0.0	
	*1. no	-6.4	2.9	1.0		*1. considerably and slightly lower skilled	-10.3	1.8	-4.3	0.047 (1 VS 2)	*1. considerably and slightly lower skilled	-6.7	1.3	-1.4		*1. thin	-8.3	1.6	-7.1	
	*2. neither	-6.4	1.3	1.1		*2. slightly highly skilled	-5.8	1.5	0.2		*2. slightly highly skilled	-8.3	1.2	-3.1		*2. maintain the present body	-7.3	1.0	-6.2	
	*3. yes	-7.5	1.2	0.0		*3. considerably highly skill	-6.0	1.2	0.0	0.042 (1 VS 3)	*3. considerably highly skill	-5.2	1.4	0.0		*3. overweight or obese	-1.1	2.9	0.0	
Non-ambulatory	1. no	-0.6	1.7	1.5		1. considerably and slightly lower skilled	-3.0	1.1	-0.4		1. considerably and slightly lower skilled	-4.0	0.9	-2.4	0.024 (1 VS 2)	1. thin	-1.8	1.1	1.6	
	2. neither	-3.2	0.8	-1.1		2. slightly highly skilled	-1.7	0.9	0.8		2. slightly highly skilled	-1.4	0.8	0.2		2. maintain the present body	-2.3	0.7	1.1	
	3. yes	-2.1	0.7	0.0		3. considerably highly skill	-2.5	0.8	0.0		3. considerably highly skill	-1.6	1.0	0.0		3. overweight or obese	-3.4	2.1	0.0	
	*1. no	-0.6	1.7	1.6		*1. considerably and slightly lower skilled	-3.0	1.1	-0.5		*1. considerably and slightly lower skilled	-3.9	0.9	-2.4	0.033 (1 VS 2)	*1. thin	-1.8	1.1	1.4	
	*2. neither	-3.2	0.8	-1.1		*2. slightly highly skilled	-1.8	0.9	0.7		*2. slightly highly skilled	-1.4	0.8	0.1		*2. maintain the present body	-2.3	0.7	0.9	
	*3. yes	-2.1	0.7	0.0		*3. considerably highly skill	-2.5	0.8	0.0		*3. considerably highly skill	-1.5	1.0	0.0		*3. overweight or obese	-3.2	2.1	0.0	
Total	1. no	-7.1	3.7	2.4		1. considerably and slightly lower skilled	-13.6	2.3	-5.1	0.041 (1 VS 2)	1. considerably and slightly lower skilled	-11.1	1.7	-4.7		1. thin	-10.0	2.2	-5.1	
	2. neither	-9.7	1.7	-0.1		2. slightly highly skilled	-7.5	1.9	1.0		2. slightly highly skilled	-9.6	1.6	-3.2		2. maintain the present body	-9.6	1.4	-4.7	
	3. yes	-9.5	1.6	0.0		3. considerably highly skill	-8.5	1.6	0.0		3. considerably highly skill	-6.4	1.9	0.0		3. overweight or obese	-4.9	4.1	0.0	
	*1. no	-6.6	3.7	2.9		*1. considerably and slightly lower skilled	-13.3	2.3	-5.1		*1. considerably and slightly lower skilled	-10.7	1.7	-4.3		*1. thin	-10.1	2.2	-5.9	
	*2. neither	-9.6	1.7	-0.1		*2. slightly highly skilled	-7.8	1.9	0.5		*2. slightly highly skilled	-9.7	1.6	-3.3		*2. maintain the present body	-9.5	1.3	-5.2	
	*3. yes	-9.5	1.6	0.0		*3. considerably highly skill	-8.3	1.6	0.0		*3. considerably highly skill	-6.4	1.9	0.0		*3. overweight or obese	-4.3	4.1	0.0	
ΔMPA (min/day)																				
Ambulatory	1. no	-5.6	2.6	0.1		1. considerably and slightly lower skilled	-8.9	1.6	-4.6	0.025 (1 VS 2)	1. considerably and slightly lower skilled	-5.4	1.2	-1.8		1. thin	-6.6	1.5	-6.6	
	2. neither	-4.8	1.2	1.0		2. slightly highly skilled	-4.2	1.3	0.1		2. slightly highly skilled	-6.4	1.1	-2.8		2. maintain the present body	-5.6	0.9	-5.6	0.047 (2VS3)
	3. yes	-5.7	1.2	0.0		3. considerably highly skill	-4.4	1.1	0.0	0.020 (1 VS 3)	3. considerably highly skill	-3.7	1.3	0.0		3. overweight or obese	0.0	2.7	0.0	
	*1. no	-5.2	2.7	0.6		*1. considerably and slightly lower skilled	-8.8	1.6	-4.5	0.037 (1 VS 2)	*1. considerably and slightly lower skilled	-5.2	1.2	-1.4		*1. thin	-6.7	1.5	-7.0	
	*2. neither	-4.8	1.2	1.0		*2. slightly highly skilled	-4.4	1.4	-0.1		*2. slightly highly skilled	-6.5	1.1	-2.7		*2. maintain the present body	-5.7	0.9	-6.0	0.047 (2VS3)
	*3. yes	-5.8	1.2	0.0		*3. considerably highly skill	-4.3	1.1	0.0	0.024 (1 VS 3)	*3. considerably highly skill	-3.8	1.3	0.0		*3. overweight or obese	0.3	2.7	0.0	
Non-ambulatory	1. no	-0.6	1.7	1.3		1. considerably and slightly lower skilled	-2.8	1.1	-0.5		1. considerably and slightly lower skilled	-3.7	0.8	-2.5	0.021 (1 VS 2)	1. thin	-1.7	1.1	1.3	
	2. neither	-2.8	0.8	-1.0		2. slightly highly skilled	-1.5	0.9	0.7		2. slightly highly skilled	-1.2	0.8	0.1		2. maintain the present body	-1.9	0.6	1.1	
	3. yes	-1.9	0.7	0.0		3. considerably highly skill	-2.2	0.7	0.0		3. considerably highly skill	-1.2	0.9	0.0	0.044 (1 VS 3)	3. overweight or obese	-3.0	2.0	0.0	
	*1. no	-0.5	1.7	1.3		*1. considerably and slightly lower skilled	-2.7	1.1	-0.5		*1. considerably and slightly lower skilled	-3.7	0.8	-2.4	0.028 (1 VS 2)	*1. thin	-1.7	1.1	1.2	
	*2. neither	-2.8	0.8	-1.0		*2. slightly highly skilled	-1.6	0.9	0.6		*2. slightly highly skilled	-1.2	0.8	0.0		*2. maintain the present body	-2.0	0.6	0.9	
	*3. yes	-1.9	0.7	0.0		*3. considerably highly skill	-2.2	0.7	0.0		*3. considerably highly skill	-1.2	0.9	0.0	0.049 (1 VS 3)	*3. overweight or obese	-2.9	2.0	0.0	
Total	1. no	-5.8	3.4	1.7		1. considerably and slightly lower skilled	-11.1	2.1	-4.7		1. considerably and slightly lower skilled	-9.0	1.6	-4.2		1. thin	-8.1	2.0	-5.2	
	2. neither	-7.4	1.6	0.2		2. slightly highly skilled	-5.9	1.8	0.5		2. slightly highly skilled	-7.5	1.5	-2.8		2. maintain the present body	-7.5	1.2	-4.6	
	3. yes	-7.6	1.5	0.0		3. considerably highly skill	-6.4	1.5	0.0		3. considerably highly skill	-4.7	1.8	0.0		3. overweight or obese	-2.9	3.7	0.0	
	*1. no	-5.4	3.4	2.1		*1. considerably and slightly lower skilled	-11.0	2.1	-4.8		*1. considerably and slightly lower skilled	-8.6	1.6	-3.8		*1. thin	-8.2	2.0	-6.0	
	*2. neither	-7.4	1.6	0.2		*2. slightly highly skilled	-6.1	1.8	0.1		*2. slightly highly skilled	-7.6	1.5	-2.8		*2. maintain the present body	-7.5	1.2	-5.3	
	*3. yes	-7.5	1.5	0.0		*3. considerably highly skill	-6.2	1.5	0.0		*3. considerably highly skill	-4.8	1.7	0.0		*3. overweight or obese	-2.2	3.7	0.0	
ΔStep count (steps/day)	1. no	-2021	668	-290		1. considerably and slightly lower skilled	-2563	415	-1077	0.023 (1 VS 2)	1. considerably and slightly lower skilled	-1968	325	-827		1. thin	-1902	419	-1027	
	2. neither	-1495	313	236		2. slightly highly skilled	-1352	347	135		2. slightly highly skilled	-1807	306	-666		2. maintain the present body	-1682	256	-806	
	3. yes	-1731	294	0		3. considerably highly skill	-1487	288	0	0.031 (1 VS 3)	3. considerably highly skill	-1141	353	0		3. overweight or obese	-875	781	0	

LPA: Light physical activity, MVPA: moderate-to-vigorous physical activity, MPA: moderate physical activity, SE: standard error, Δ: change, Δvariables were calculated as follow-up values minus baseline values, adjusted for school, follow-up periods, age and sedentary behavior or physical activity at baseline, *: adjusted for school, follow-up periods, age and sedentary behavior or moderate-to-vigorous physical activity at baseline.
